# Long-term risks of invasive cervical cancer following HPV infection: follow-up of two screening cohorts in Manchester

**DOI:** 10.1038/s41416-023-02227-9

**Published:** 2023-03-23

**Authors:** Clare Gilham, Alexandra Sargent, Emma J. Crosbie, Julian Peto

**Affiliations:** 1 London School of Hygiene & Tropical Medicine, London, UK; 2 Cytology Department, Manchester Royal Infirmary, Manchester University NHS Foundation Trust, Manchester, UK; 3 Division of Gynaecology, Manchester University NHS Foundation Trust, Manchester Academic Health Science Centre, Manchester, UK; 4 Division of Cancer Sciences, University of Manchester, Faculty of Biology, Medicine and Health, Manchester, UK

**Keywords:** Epidemiology, Cancer screening

## Abstract

**Background:**

Long-term follow-up of large cohorts is needed to determine the effects of HPV and screening on CIN3 (grade 3 cervical intraepithelial neoplasia) and ICC (invasive cervical cancer).

**Methods:**

Women were recruited when attending for routine cervical screening in Greater Manchester, UK: 1987–93 for the Manchester Cohort (MC: 47,625 women) and 2001–03 for the ARTISTIC Cohort (AC: 24,496 women). Both were followed through national registration for cancer incidence and mortality to 2020.

**Results:**

Risk patterns following HPV infection differed for CIN3 and ICC. Risk of ICC in the MC rises for 30 years following a single positive HPV test, reaching 2.5% (95% CI: 1.3–4.5%). A similar pattern was seen in the AC, but the risks of cancer were approximately halved. CIN3 was diagnosed much sooner in the AC due to more efficient cytology. More sensitive HPV testing was able to better predict future risk.

**Conclusion:**

The sensitivity of HPV testing and cytology influences the CIN3 detection rate. Sensitive HPV testing enables effective risk stratification. Increased risk of ICC is observed 15–30 years after HPV infection. Women testing HPV + should be followed until their infection clears. Discharging women from screening programmes whilst they remain HPV + may not be safe, even if cytology and colposcopy tests are normal.

## Background

Over the past 30 years, cervical cancer incidence and mortality in the UK have declined considerably as a result of a highly effective cervical screening programme (CSP) despite increasing HPV infection rates [1, 2]. Primary HPV testing is more effective than primary cytology screening [3], but the NHS (National Health Service) CSP is facing new challenges since primary HPV screening was introduced in 2019 including the management of HPV-positive cytology triage negative women. The large pilot of primary HPV screening in England has shown increased detection of high-grade cervical intraepithelial neoplasia (CIN2 + ) compared to primary cytology screening followed by lower incidence at the next screening round [4] and supports the extension of screening intervals following a negative HPV test [5]. The rarity of invasive cervical cancer (ICC) means that most studies are not large enough to study cancer as an outcome and are obliged to report CIN2 + or CIN3 + as a surrogate. Long-term follow-up of large cohorts is therefore needed to complete our understanding of lifetime cancer risks following HPV infection. The Manchester Cohort (MC) was initiated in 1987, around the time of the launch of the NHSCSP where nationally, all women aged between 20 and 64 years were invited for screening every 3–5 years, and provides the longest follow-up data in the world on cancer incidence following HPV testing (on stored samples). The ARTISTIC trial cohort (AC) recruited in a similar geographical area in 2001–03 following the introduction of liquid-based cytology (LBC), and provides 17 years of follow-up.

## Methods

### The Manchester Cohort (MC)

Between 1987 and 1993, in collaboration with over 100 general practitioners and screening clinics in the Greater Manchester area, 78,062 cervical samples were collected from 61,564 women attending for routine Papanicolaou (pap) screening. There was no age restriction. Women consented to having a cervical sample taken and stored after their routine smear. Samples were stored at −30 °C [6]. After the initial sample was collected, participants were not contacted, but were followed passively. The women were managed according to local cervical screening guidelines, for those still residing in Manchester: received invitations to attend for Pap smears every 5 years until 2004, then liquid-based cytology every 3 years (5 yearly if aged >50 years), with the addition of triage of low-grade cytology with HPV testing from 2008, and then finally primary HPV testing from 2019 (or from 2013 if in pilot areas).

To avoid the cost of testing all stored samples for HPV DNA using PCR, an age-stratified random sample of 7278 (11.8%) women (aged 15–69) was selected for testing in the early 1990s. HPV L1 consensus PCR amplification was used with MY09/MY11 primers [7, 8]. An internal β-globin control indicated adequacy among 89% (*n* = 6462) of the samples assayed. Samples found to be HPV-positive were dot blotted onto new membranes and hybridised with a series of biotinylated high-risk HPV (HR-HPV) type-specific probes. Further description of the sample collection methods and HPV results have been published elsewhere [6].

**Fig. 1:**
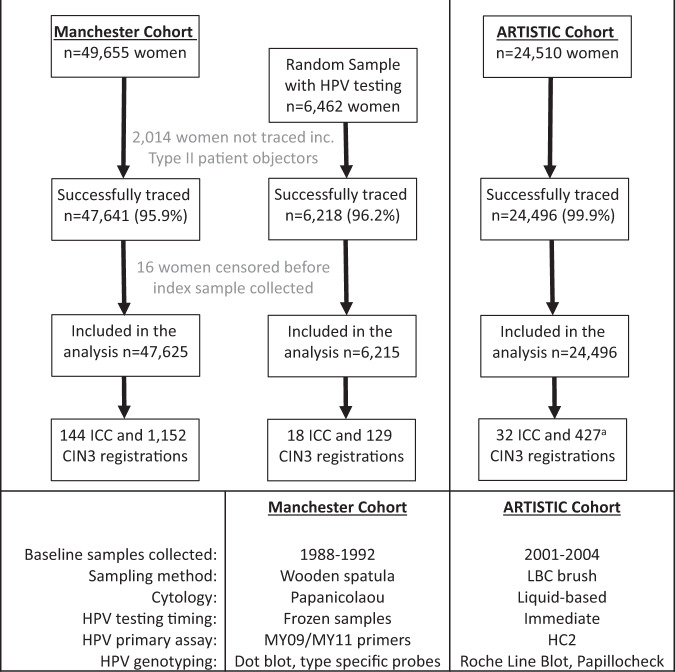
Consort diagram showing total number of women analysed in the cohorts, including those randomly selected for HPV testing in the Manchester Cohort. ^a^Three women had both CIN3 and ICC registrations on the same day, so are classified as ICC in cumulative risk analysis.

Samples were excluded if they were taken before July 1988 due to storage procedure (*n* = 7504), inadequate Pap smear (*n* = 3391), abnormal smear within the previous year (*n* = 509), or they had ever had a diagnosis of CIN3 (*n* = 505). All previous analyses were based on the remaining 49,655 women from whom a sample for HPV assay was collected at a routine smear test that gave an adequate cytological result [6]. These 49,655 women were traced through the NHS Central Register until May 2020 for mortality and cancer incidence including CIN3 (cervical cancer in-situ) and 47,641 (96%) were successfully flagged. Sixteen women were censored before the baseline sample leaving 47,625 in the cohort, including 6215 in the random sample whose baseline samples were tested for HPV (Fig. 1).

### The ARTISTIC Trial cohort (AC)

Total of 24,510 women aged 20–64 were recruited from a similar area of Greater Manchester between 2001 and 2003 to take part in a trial of HPV testing compared to cytology screening (LBC). All LBC samples taken (as part of the NHSCSP) from women in the cohort were tested for HPV until 2009. Women were randomised in a 3:1 ratio to have their HPV result revealed. Women with abnormal cytology were managed according to current guidelines and hence the only difference between the arms of the trial was among women with normal cytology, where women with HPV were offered repeat HPV testing and if persistent, colposcopy. Hybrid Capture 2 (HC2) was used as the primary HPV assay with HC2-positive and a sample of HC2-negative samples further tested with PCR-based assays (Roche Line Blot, Papillocheck) [9]. Samples testing HC2-positive but with no HR-HPV DNA detected by a PCR test and HC2-negative samples were all assumed HR-HPV-negative. A small number of samples (*n* = 24) that tested HC2-positive but were insufficient for further typing were assumed to be HR-HPV-positive. The cohort has been followed via national registration for cancer incidence and mortality (date only, cause not known) until May 2020 resulting in 24,496 women contributing a total of 409,094 person-years (Fig. 1). The cohort was also followed up through the histology laboratories until the end of 2009 which yielded a further 96 laboratory records of CIN3 which were not recorded in a cancer registry during the first 8 years of the trial [9]. These analyses are restricted to registered cases on which national rates are based. Three CIN3 diagnoses were categorised as ICC only for the cumulative risk analysis because they had cervical cancer registrations on the same date (Fig. 1).

### Statistical analysis

Women from the random sample in the Manchester cohort (MC) and all women from the ARTISTIC cohort (AC) were classified hierarchically into three mutually exclusive groups: HPV16 or HPV18, any other HR-HPV (types 31, 33, 35, 39, 45, 51, 52, 56, 58, 59 and 68) without HPV16 or HPV18, and HPV-negative. Those testing positive for types other than these 13 HR types were defined as HR-HPV-negative. Cumulative cancer and CIN3 risks were estimated by Kaplan–Meier methods using the date of cancer registration as the outcome with censoring at the date of emigration, date of death, age 85 years, and on March 31, 2019 to allow for late registration of CIN3 and cancer. For the CIN3 analysis, women were censored at age 70, rather than age 85, as they are rarely screened after this age.

Total person-years were calculated and follow-up time was stratified by time period (in 5-year periods from 1987 for the MC and 2002 for the AC) and age group (in 5-year age groups from age 15 to age 69 for CIN3 and age 84 for cancer). English national cancer incidence rates and mortality rates for England and Wales were used to calculate standard incidence ratios (SIRs) and standard mortality ratios (SMRs), respectively, for selected gynaecological and other common cancers (cervix, cervical cancer in-situ, vulva, vagina, ovary, anus, oropharynx, lung, breast and colon). Cervical cancer incidence rates are around 50% higher in the NW (northwest) than in the London (southeast) region of England and about 20% higher than the average for England. There is a similar north/south divide regarding lung cancer incidence, which is around 35% higher in the NW than in England as a whole. Rates in the NW are similar to national rates for breast, vagina, vulva, anus, ovarian and colon cancers [10]. For the incidence analyses, women were not censored at first cancer registration to allow for subsequent cancer registrations but second cancers at the same site were ignored. Poisson regression was used to estimate rate ratios comparing those with and without HPV infection at baseline adjusted for age and period. All analyses were programmed in STATA 17.0 (Stata Corp 2021).

## Results

Within 30 years of follow-up invasive cervical cancer (ICC) was diagnosed in 144 of the 47,625 women in the MC, 35 of whom died with cervical cancer as the stated cause. Among the 24,496 women in the AC, 32 were diagnosed with ICC over 17 years of follow-up. Table 1 compares incidence in the cohorts from the four cancers most strongly associated with HPV infection (cervix, anus, vagina and vulva) [11] with the national population rates for England and Wales. The incidence of cervical cancer was 11% lower than national rates in the MC (SIR = 0.89, 95% CI: 0.76–1.05) and 36% lower in the AC (SIR = 0.64, 95% CI: 0.45–0.91), and cervical cancer mortality was substantially reduced in the MC (SMR = 0.67, 95% CI: 0.48–0.94: Supplementary Table 2). SIRs for cancers of the anus, vagina and vulva were also lower in the AC. The SIR for CIN3 was 1.11 (95% CI: 1.01–1.23) in the AC and 1.02 (95% CI: 0.97–1.09) in the MC.

**Table 1:** Cancer incidence rates, standard incidence rates (SIR) and adjusted rate ratios (RR) following HPV status at baseline for women in the Manchester and ARTISTIC Cohorts.

	*N* cancer registrations	Cancer incidence rate per 1000	SIR	*N* cancer registrations			Adjusted RR^a^	*P* value
	Manchester Cohort of 47,625 women				HPV-positive (*n* = 425)	HPV-negative (*n* = 5790)		
Cervix	143^b^	0.11 (0.10–0.13)	0.89 (0.76–1.05)	10		8	19.61 (7.12–53.97)	<0.0001
CIN3	1152^c^	1.01 (0.96–1.07)	1.02 (0.97–1.09)	49		80	5.87 (4.03–8.54)	<0.0001
Vulva	39	0.03 (0.02–0.04)	0.95 (0.69–1.30)	1		3	4.68 (0.42–51.78)	0.3
Vagina	8	0.01 (0.00–0.01)	0.82 (0.41–1.65)	0		0		
Anus	29	0.02 (0.02–0.03)	1.01 (0.70–1.45)	1		3	5.02 (0.49–51. 97)	0.2
	ARTISTIC Cohort of 24,496 women				HPV-positive (*n* = 2804)	HPV-negative (*n* = 21,692)		
Cervix	32	0.08 (0.06–0.11)	0.64 (0.45–0.91)	20		12	16.06 (7.41–34.78)	<0.0001
CIN3	427^d^	1.10 (1.00–1.20)	1.11 (1.01–1.23)	337		90	17.72 (13.58–22.67)	<0.0001
Vulva	10	0.02 (0.01–0.05)	0.76 (0.41–1.40)	2		8	4.02 (0.78–20.75)	0.1
Vagina	1	0.00 (0.00–0.02)	0.32 (0.05–2.30)	0		1		
Anus	3	0.01 (0.00–0.02)	0.26 (0.08–0.81)	1		2	3.82 (0.23–62.54)	0.4

Women are censored at age 85 years for cancer and age 70 for CIN3.

^a^Rate ratios calculated by Poisson regression adjusted for age group and period.

^b^One cervical cancer (diagnosed at age 88) was censored and excluded.

^c^Three CIN3s (diagnosed at ages 70, 74 and 84) were censored and excluded.

^d^Three CIN3 registrations included for those who had a concurrent cervical cancer registration. These CIN3 registrations have been ignored in subsequent tables.

Lung cancer incidence and mortality ratios were elevated in the MC (SIR = 1.31, 95% CI: 1.22–1.39 and SMR = 1.46, 95% CI: 1.36–1.57) but not in the AC (SIR = 0.96, 95% CI: 0.84–1.11, Supplementary Tables 1 and 2). Incidence and mortality rates for other common cancers were similar to general population rates in both cohorts (Supplementary Tables 1 and 2).

Internal comparisons between those with and without HPV infection at baseline are shown in Table 1. The incidence rate was much higher among women with HPV at baseline in both cohorts for invasive cervical cancer (MC RR = 19.6, AC RR = 16.1; both *P* < 0.0001) and CIN3 (MC RR = 5.9, AC RR = 17.7; both *P* < 0.0001). There were insufficient numbers of vaginal cancers to estimate rate ratios but elevated risks were seen for cancers of the vulva (MC RR = 4.7, AC RR = 4.0) and anus (MC RR = 5.0, AC RR = 3.8) in those testing HPV-positive at baseline. There was some evidence of elevated lung cancer incidence in HPV-positive women in the MC (RR = 2.3, 95% CI: 1.2–4.6, *P* = 0.03), but not in the AC (RR = 1.0) (Supplementary Table 1). Despite the small number of deaths mortality rates from cervical and anal cancer were significantly higher among those with HPV infection at baseline in the MC (RR = 11.5, 95% CI: 1.7–78.5 for cervix based on 5 deaths and RR = 19.9, 95% CI: 1.2–317.5 for anus based on two deaths, Supplementary Table 2).

**Fig. 2:**
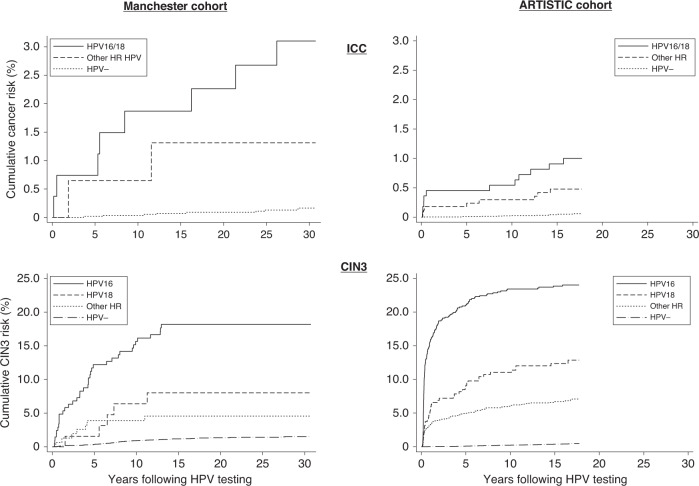
Cumulative risk of invasive cervical cancer (ICC) and CIN3 by HPV infection at baseline. The upper figures show the risk of ICC (18 ICC in the MC and 32 ICC in the AC) and the lower figures show the risk of CIN3 (129 CIN3 in the MC and 520 CIN3 in the AC).

The risk of invasive cervical cancer continued to rise for up to 30 years following a single positive HPV test (Table 2 and Fig. 2) resulting in a 30-year cumulative risk of 2.5% (95% CI: 1.3–4.5%) in the MC. For both cohorts, we observed a higher risk in women infected with HPV16 or HPV18 compared to other HR genotypes but in the AC the risks were approximately halved. The 15-year risks were 1.9% in MC and 0.9% in AC for HPV16/18, and 1.3% in MC and 0.5% in AC for other HR-HPVs (Supplementary Table 3). The 15-year cervical cancer risk following a single negative HPV test was about 50% lower than following a single negative cytology test (0.07% vs 0.15% in the MC and 0.04% vs 0.07% in the AC).

**Table 2:** Cumulative risk of invasive cervical cancer and CIN3 by HPV infection (*n* = 6215) and cytology at baseline (*n* = 47,625) in the Manchester Cohort and by HPV infection and cytology in the ARTISTIC trial cohort (*n* = 24,496); women are censored at the first occurrence of ICC, but not by age^a^.

Status at baseline			5 years from baseline			15 years from baseline			30 years from baseline		
	*n* (%) at the baseline	*n* cases^b^	*n* cases	%	95% CI	*n* cases	%	95% CI	*n* cases	%	95% CI
Manchester Cohort—ICC											
HR-HPV +	425 (6.8%)	10	3	0.71%	(0.23–2.17)	7	1.67%	(0.80–3.46)	10	2.46%	(1.33–4.52)
HR-HPV−	5790 (93.2%)	8	1^c^	0.02%	(0.00–0.12)	4	0.07%	(0.03–0.19)	8	0.16%	(0.08–0.33)
Normal cytology	45,108 (94.7%)	113	16	0.04%	(0.02–0.06)	66	0.15%	(0.12–0.19)	113	0.28%	(0.23–0.33)
Low-grade cytology	2152 (4.5%)	16	4	0.19%	(0.07–0.50)	9	0.42%	(0.22–0.81)	16	1.02%	(0.58–1.79)
High-grade cytology	364 (0.8%)	15	12	3.30%	(1.89–5.73)	14	3.87%	(2.31–6.45)	15	4.18%	(2.54–6.84)
ARTISTIC Cohort— ICC											
HR-HPV +^d^	2804 (11.4%)	20	10	0.36%	(0.19–0.66)	19	0.68%	(0.43–1.06)			
HR-HPV−^e^	21,692 (88.6%)	12	2	0.01%	(0.00–0.04)	9	0.04%	(0.02–0.08)			
Normal cytology	21,369 (87.2%)	18	2	0.01%	(0.00–0.04)	15	0.07%	(0.04–0.12)			
Low-grade cytology	2665 (10.9%)	3	0			2	0.08%	(0.02–0.30)			
High-grade cytology	462 (1.9%)	11	10	2.16%	(1.17–3.99)	11	2.38%	(1.33–4.26)			
Manchester Cohort—CIN3											
HR-HPV +	425 (6.8%)	49	32	7.57%	(5.41–10.53)	49	11.70%	(8.97–15.18)	49	11.70%	(8.97–15.18)
HR-HPV−	5790 (93.2%)	80	21	0.36%	(0.24–0.56)	66	1.17%	(0.92–1.49)	80	1.51%	(1.21–1.89)
Normal cytology	45,108 (94.7%)	866	128	0.29%	(0.25–0.35)	722	1.75%	(1.63–1.88)	866	2.21%	(2.07–2.37)
Low-grade cytology	2152 (4.5%)	200	132	6.16%	(5.22–7.26)	192	9.04%	(7.89–10.34)	200	9.46%	(8.28–10.79)
High-grade cytology	364 (0.8%)	90	85	23.45%	(19.42–28.16)	90	24.93%	(20.79–29.73)	90	24.93%	(20.79–29.73)
ARTISTIC Cohort—CIN3											
HR-HPV + ^d^	2804 (11.4%)	336	272	9.84%	(8.79–11.02)	330	12.01%	(10.85–13.29)			
HR-HPV−^e^	21,692 (88.6%)	88	21	0.10%	(0.06–0.15)	77	0.37%	(0.30–0.47)			
Normal cytology	21,369 (87.2%)	147	42	0.20%	(0.15–0.27)	134	0.65%	(0.55–0.77)			
Low-grade cytology	2665 (10.9%)	98	75	2.84%	(2.27–3.54)	94	3.57%	(2.93–4.36)			
High-grade cytology	462 (1.9%)	179	176	40.54%	(36.06–45.33)	179	41.29%	(36.79–46.12)			

^a^One cervical cancer diagnosed age 88 in the MC appears in this table but is censored in Table 1.

^b^To the end of the follow-up to March 2019 (max follow-up 30 years for MC and 17 years for AC).

^c^Tested HPV-positive with general primers but negative for HR type-specific primers.

^d^Includes 24 women who were HC2 + and insufficient for typing (includes 1 diagnosed with ICC at baseline following high-grade cytology).

^e^HC2-negative and HC2-positive but with no HR-HPV DNA detected on genotyping.

**Fig. 3:**
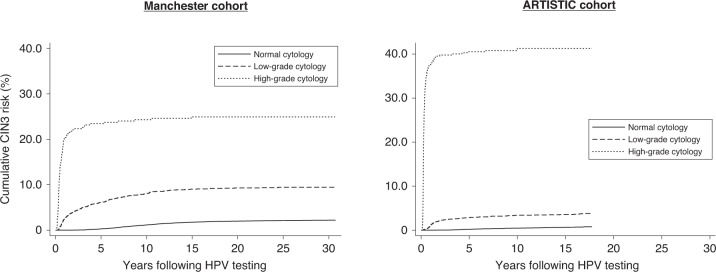
Cumulative CIN3 risk by cytology at baseline. The analysis includes 1156 CIN3 in the Manchester cohort (left hand figure) and 424 CIN3 in the ARTISTIC cohort (right hand figure).

Figure 2 shows higher cumulative CIN3 risks following HPV infection in the AC compared with the MC. In both cohorts, HPV16 conferred the highest risk (15-year risks: 18.2% in MC and 23.9% in AC, Supplementary Table 4a, b). Regardless of HPV genotype, the risk of CIN3 in the MC continues to rise for approximately 10 years following a single positive HPV test and then plateaus, with no CIN3 diagnoses beyond 13 years in women who tested HPV-positive at baseline (Table 2). The shape of the curves differ between the cohorts and show CIN3 diagnosed much sooner in the AC (the proportion of HPV-positive CIN3s diagnosed within 1, 2 and 5 years of baseline were 57%, 68% and 81% in the AC and 22%, 33% and 65% in the MC). This is partly because cytology was better able to identify those at highest risk in the AC (Table 2 and Fig. 3). Overall, 5% of women had abnormal cytology at baseline in the MC compared to 13% in the AC, and of the CIN3 diagnosed within 5 years, 57% had high-grade cytology at baseline in the AC compared to 25% in the MC. Figure 3 shows similar shaped curves between the cohorts once stratified by cytology, but a higher sensitivity of high-grade cytology (5-year CIN3 risk of 41% in AC compared to 23% in the MC). Among women with normal cytology at baseline the 5 year cumulative CIN3 risks were similar (0.29% in the MC and 0.20% in the AC) but the 15-year risk was much higher in the MC (1.75% compared to 0.65% in the AC).

**Fig. 4:**
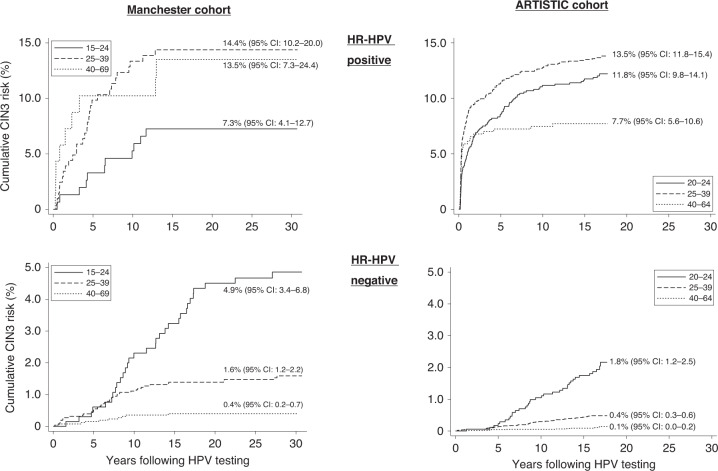
Cumulative CIN3 risk by age at baseline. HR-HPV positive women at baseline are shown in upper figures and in HR-HPV negative women at baseline in lower figures. Cumulative risks with 95% Confidence Intervals are shown on the graphs at 30 years and 15 years respectively for the Manchester and ARTISTIC Cohorts.

Figure 4 shows CIN3 risks by age group and HPV status at baseline. Among 2804 HPV-positive women in the AC there was a lower long-term CIN3 risk among women aged over 40, but this was not seen among the 425 HPV-positive women in the MC (Fig. 4). The risk of CIN3 in both cohorts continued to increase among women testing HPV-negative at baseline, and was highest among the youngest women (Fig. 4). In HPV-negative women, 15-year CIN3 risks were higher for the MC compared to the AC: 2.8% vs 1.8% in 20–24-year-olds, 1.4% vs 0.4% in 25–39-year-olds and 0.4% vs 0.1% in ≥40-year-olds (Fig. 4 and Supplementary Table 4a, b).

## Discussion

### Sensitivity of cytology and HPV testing

Samples for HPV testing and cytology were collected about 12 years apart in the two cohorts (1987–93 in the MC and 2001–03 in the AC). Referral criteria based on cytology alone were similar in both cohorts except for HPV-infected women in the AC with normal cytology, but the modern sampling and cytology methods employed by the ARTISTIC trial (summarised in Fig. 1) resulted in higher sensitivity for CIN3 + detection following both cytology and HPV testing. Women diagnosed with CIN3 in the first 5 years of follow-up were much more likely to test HPV-positive and/or have abnormal cytology at baseline in the AC (93% HPV + and 86% abnormal cytology in the AC vs 60% and 63% in the MC). Both HPV testing and cytology were, therefore, much more effective in stratifying women in the AC in order to predict future risk of both CIN3 and ICC.

### HPV-positive women and sensitivity of cytology

Respective 15-year cumulative risks of CIN3 in MC and AC were 11.7% and 12.0% following any HR-HPV infection and 18.2% and 23.9% following HPV16 infection. Among women positive at baseline, CIN3 was diagnosed much earlier in the AC (68% vs 33% of CIN3 diagnosed within 2 years in AC and MC, respectively). Due to the referral procedures, the majority of CIN3s were diagnosed following abnormal cytology in both cohorts, but only 16% (8/49) of CIN3s in the MC had high-grade cytology at baseline, compared to 52% (175/336) in the AC. The pattern of cumulative risk of CIN3 (lower graph of Fig. 2) in the AC shows that CIN3 usually develops within a few years of HPV infection [12], although diagnosis was often delayed for several years with the less sensitive Pap cytology in the MC. The 5-year cumulative CIN3 risk following normal cytology was similar in the cohorts, but much higher in the MC after 15 years (MC: 1.8%, 95% CI: 1.6–1.9 and AC: 0.7%, 95% CI: 0.6–0.8) further indicating that cytology in the MC was less sensitive. Other studies have not shown an increased sensitivity of LBC when directly compared to conventional Pap cytology in parallel [13]. LBC was rolled out to the women participating in the AC ahead of the rest of the local area which involved staff retraining which may explain the increased sensitivity, as lesions previously missed by conventional cytology were detected with the first LBC test [14]. The 15-year cumulative incidence of ICC following HPV infection was approximately twice as high in the MC compared to the AC (1.7% vs 0.7%). The SIRs (Table 1) show the CIN3 and ICC incidences in the MC were similar to the general population at the time, but the AC experienced better screening than the general population with ~10% higher incidence of CIN3 and 40% less ICC. This may be due to a combination of better laboratory performance and selection bias. Both cohorts allowed recruitment of all women attending screening for any reason (routine recall or early recall following abnormality), but the AC women were recruited to a randomised trial and therefore were likely to be more selected than the women participating in the MC.

Many natural history models are based on a CIN3 + endpoint due to the rarity of ICC, but Fig. 2 shows very different patterns of incidence for CIN3 and ICC following HPV infection. HPV detection, particularly with HPV16/18, continues to predict a high ICC risk 15–30 years later when CIN3 is rarely diagnosed. Women testing positive for HPV infection should therefore be regularly re-screened until the infection clears. In England, one of the options proposed for primary screening is for women aged over 65 who test HPV-positive but show no abnormalities from cytology or colposcopy to exit the screening programme. In Denmark, nearly half of the women aged 60–64 did not receive adequate follow-up after a positive HPV test [15]. Our findings suggest that this is not safe.

### HPV positivity and age

HPV infections in older women may be intermittently detectable latent infections that are likely to have low risk of progression [16], but lower risks among HPV-positive women aged over 40 in the AC may also be due to better lifetime screening. English cervical CIS (CIN3) rates in women aged 40–54 almost halved from 61.6 per 100,000 in 1988 to 37.1 per 100,000 in 2001 [17], suggesting that CIN3 diagnosed in older women in 1988 had often been present for many years. By 2001, women aged over 30 had been screened for at least 10 years and the majority of prevalent CIN3s had already been diagnosed [17].

### HPV-negative women and sensitivity of HPV testing

The cumulative ICC risk at 15 years was 0.04% (95% CI: 0.02–0.08) in 21,692 HPV-negative women in the AC. The higher risk for women who tested HPV-negative in the MC is likely to be due to false negative results due to a combination of poor sample quality and lower test sensitivity. Sample collection in the MC was sub-optimal by today’s standards: a wooden spatula was used, the tip of which was broken off and stored in the sample medium before being removed sometime later [6]. In addition, the samples underwent testing at a time when HPV PCR testing was likely to have been less sensitive than the HC2 assay used in the AC.

The woman diagnosed with ICC within 5 years of a negative HPV test in the MC (footnote 3, Table 2) actually tested HPV-positive with the general HPV primers but HR types were not detected. Both the two ICCs diagnosed within 5 years of testing HPV-negative at baseline of the AC and six of seven later cancers (3 were untested) were found to contain HR-HPV DNA after more sensitive testing [9]. This suggests that a much longer screening interval would be safe following a negative HPV test with a sensitive assay, but it is not known how the more modern assays currently used by the NHS would have performed. Cancers missed by primary HPV testing cannot be investigated without a biobank of stored cervical samples, which has not yet been established in England. The UK pilot project has been linked to national registration and reported 14 interval cancers within ~3 years of a negative HPV test among approximately 350,000 women aged 24–64 [5], similar to our estimate (1 cancer within 3 years among 21,692 women). Increasing the sensitivity of a primary HPV test would detect more early cancers and give more reassurance to those testing negative, but would create further challenges because a more specific triage test would be needed to reduce referrals and anxiety. One strategy would be to increase the sensitivity of the final test when women stop being screened.

The higher long-term CIN3 risk in younger women negative for HPV at baseline reflects the pattern of acquisition of new HPV infections [9]. After a single negative HPV test in the AC, women aged over 40 had a very low future CIN3 risk (0.09% after 15 years) and the overall 5-year CIN3 risk in HPV-negative women was 0.10% (95% CI: 0.06–0.15) compared to a 5-year risk of 0.20% (95% CI: 0.15–0.27) in women following normal cytology. This provides evidence for extending screening intervals between HPV tests in the NHSCSP. However, these and other analyses [18, 19] should be regarded with some caution. The absolute risk of disease after a longer screening interval will be underestimated, as women in these evaluation studies continued to be screened and referred on the basis of cytology every 3–5 years during the follow-up.

### Non-cervical cancer incidence and mortality

Despite the rarity of other HPV-related cancers, we showed strong elevated risks of vulva and anal cancers associated with cervical HPV infection. There were insufficient numbers of vaginal and oropharyngeal cancers to explore the relationship with HPV. We observed an overall increased risk of lung cancer in the MC both compared with national rates (SIR 1.3, 95% CI: 1.2–1.4) and following HPV positivity (RR = 2.3, 95% CI: 1.2–4.6, *P* = 0.03), but not in the AC (SIR 1.0, RR = 1.0).

The overall excess presumably reflects the high local rates, but HPV has been suggested as a risk factor for lung cancer. HPV DNA has been identified in lung cancer biopsies [20], including from non-smokers [21], but few studies appear to fully adjust for confounding factors. The British National Survey of Sexual Attitudes and Lifestyles (Natsal-3) showed that increased deprivation and smoking were associated with HR-HPV positivity and non-attendance for cervical screening [22]. Previous analyses of epidemiological data from the MC and AC have not found an association between HPV positivity and smoking [23, 24], but the women taking part in these nested surveys were likely to be selected subsets of our screened cohorts. When the Poisson model shown in supplementary table 1 was adjusted for area-level deprivation, the adjusted RR for HPV infection reduced to 2.0 (95% CI: 1.0–4.0; *P* = 0.07) for the MC, indicating some confounding by deprivation.

### Strengths and limitations

The main strength of these analyses is the almost complete follow-up over 30 years for the MC and 17 years for the AC. The MC samples were collected before HPV testing techniques had been developed and the samples were processed and stored in sub-optimal conditions, but HPV-positive results are likely to be reliable despite the potentially lower sensitivity for detecting HPV infection. Only an eighth of the entire MC were originally tested for HPV infection, so a nested-case control study is currently underway in which stored samples from women who later developed ICC and other HPV-related cancers are being tested by modern sensitive PCR. Samples stored from the AC are much better quality, but were originally tested with HC2 which may not be comparable to modern HPV assays. HR-HPV was not detected in 27% of the HC2-positive samples after genotyping [9].

To maximise the numbers included in the analysis, we included 65 women aged under 20 with HR-HPV and 262 who were HR-HPV-negative in the MC analyses. They appear separately in Table 2 and Supplementary Table 3. They are likely to be a highly selected group but their cumulative risk did not differ from those aged 20–24.

The women participating in the AC consented to be randomised into a trial, and were probably more selected than those participating in the MC, who consented for their samples to be used for research. Any social class bias might account for the lower lung and cervical cancer rates seen in the AC. More regular attendance for women in the AC would also partly explain their higher CIN3 rates.

There is a degree of under-registration for carcinoma-in-situ. In order to compare the cohorts, we restricted the analysis to CIN3 diagnoses with national registration. Including the additional 96 CIN3s with no cancer registration recorded by the local pathology laboratory during the first 8 years of the AC [9] increased the 15-year cumulative risks from 12.0% to 14.8% in HR-HPV + and from 0.37 to 0.44% in HR-HPV− women.

## Conclusions

HPV infections, particularly HPV types 16 and 18, continue to confer a higher risk of invasive cervical cancer 15–30 years after infection. Women testing HPV-positive should therefore be followed until they clear their infection, and it is unsafe to discharge women from screening programmes if they remain HPV-positive even if they have negative cytology and colposcopy. By comparing these two cohorts, we have shown how the sensitivity of cytology influences the rate at which CIN3 is detected and that more sensitive HPV testing predicts future risk of CIN3 and ICC more accurately. The aim of the screening programme is to stratify women into groups reflective of their future risk of CIN3 and ICC. Those with minimal risk can be screened less often, while those at the highest risk can be screened more frequently to prevent ICC or diagnose it at an earlier stage. Sensitive HPV testing should be considered in primary screening, provided that appropriate triage testing is used to distinguish transient and persistent HPV infections.

## Supplementary information

Supplementary Material

## Data Availability

Follow-up data on women in these cohorts was provided by NHS Digital. We are not permitted to share these data.
