# Supplementary material for: Long-term risks of invasive cervical cancer following HPV infection: follow-up of two screening cohorts in Manchester
Source: Br J Cancer. 2023 Mar 23;128(10):1933–40. doi: 10.1038/s41416-023-02227-9 (PMC10147679; doi:10.1038/s41416-023-02227-9)
Supplement: Supplementary file 1 — Supplementary Material [file 41416_2023_2227_MOESM1_ESM.docx]

**Supplementary Table 1: Cancer incidence rates, Standard Incidence Rates (SIR) and adjusted Rate Ratios (RR) following HPV status at baseline for women in the Manchester and ARTISTIC Cohorts. Women are censored at age 85 years.**

|  | **N cancer registrations** | **Cancer incidence rate per 1,000** | | **SIR** | | **N cancer registrations** | **N cancer registrations** | **Adjusted RR^1^** | **P value** |
| --- | --- | --- | --- | --- | --- | --- | --- | --- | --- |
|  | **Manchester Cohort of 47,625 women** | | | | | **HPV positive**  **(n=425)** | **HPV negative**  **(n=5790)** |  |  |
| **Oropharynx** | 25 | | 0.02 (0.01-0.03) | | 1.06 (0. 72-1.57) | 0 | 4 |  |  |
| **Ovary** | 274 | | 0.22 (0.20-0.25) | | 0.91 (0.80-1.02) | 2 | 32 | 1.19 (0.28-5.09) | 0.8 |
| **Colon** | 422 | | 0.34 (0.31-0.37) | | 1.03 (0.94-1.14) | 0 | 69 |  |  |
| **Lung** | 874 | | 0.70 (0.66-0.75) | | 1.31 (1.22-1.39) | 9 | 126 | 2.30 (1.16-4.56)^2^ | 0.03 |
| **Breast** | 2,228 | | 1.79 (1.71-1.86) | | 0.97 (0.93-1.01) | 16 | 336 | 0.94 (0.57-1.56) | 0.8 |
|  | **ARTISTIC Cohort of 24,496 women** | | | | | **HPV positive (n=2,804)** | **HPV negative (n=21,692)** |  |  |
| **Oropharynx** | 14 | | 0.03 (0.02-0.06) | | 1.29 (0.77-2.19) | 0 | 14 |  |  |
| **Ovary** | 88 | | 0.22 (0.17-0.27) | | 0.95 (0.77-1.17) | 4 | 84 | 0.69 (0.25-1.92) | 0.5 |
| **Colon** | 106 | | 0.26 (0.21-0.31) | | 0.94 (0.78-1.13) | 6 | 100 | 1.23 (0.54-2.85) | 0.6 |
| **Lung** | 191 | | 0.47 (0.41-0.54) | | 0.96 (0.84-1.11) | 8 | 183 | 1.04 (0.51-2.14) | 0.9 |
| **Breast** | 793 | | 1.94 (1.81-2.08) | | 0.98 (0.92-1.05) | 46 | 747 | 0.93 (0.69-1.26) | 0.6 |

^1^ Rate ratios calculated by Poisson regression adjusted for age group and period

^2^ RR=2.01 (95%CI: 1.01-4.00), p=0.07 after additionally adjusting for area-level index of deprivation

**Supplementary Table 2: Cancer mortality rates, Standard Mortality Rates (SMR) and adjusted Rate Ratios (RR) following HPV status at baseline for women in the Manchester Cohort. Women are censored at age 85 years.**

|  | **N cancer deaths** | **Cancer mortality rate per 1,000** | | **SMR** | | **N cancer deaths** | **N cancer deaths** | **Adjusted RR^1^** | **P value** |
| --- | --- | --- | --- | --- | --- | --- | --- | --- | --- |
|  | **Manchester Cohort of 47,625 women** | | | | | **HPV positive**  **(n=425)** | **HPV negative**  **(n=5790)** |  |  |
| **Cervix** | 35 | | 0.028 (0.020-0.039) | | 0.67 (0.48-0.94) | 2 | 3 | 11.51 (1.68-78.48) | 0.03 |
| **Vulva** | 12 | | 0.010 (0.006-0.017) | | 1.22 (0.69-2.15) | 0 | 1 |  |  |
| **Vagina** | 5 | | 0.004 (0.002-0.010) | | 1.50 (0.62-3.60) | 0 | 0 |  |  |
| **Anus** | 11 | | 0.009 (0.005-0.016) | | 1.79 (0.99-3.23) | 1 | 1 | 19.86 (1.24-317.49) | 0.2 |
| **Oropharynx** | 1 | | 0.001 (0.000-0.006) | | 0.19 (0.03-1.32) | 0 | 0 |  |  |
| **Ovary** | 167 | | 0.134 (0.115-0.156) | | 0.96 (0.82-1.11) | 1 | 21 | 1.14 (0.15-8.67) | 0.9 |
| **Colon** | 157 | | 0.126 (0.108-0.147) | | 1.07 (0.92-1.25) | 1 | 20 | 1.74 (0.23-13.25) | 0.6 |
| **Lung** | 758 | | 0.607 (0.566-0.652) | | 1.46 (1.36-1.57) | 6 | 101 | 2.02 (0.88-4.65) | 0.1 |
| **Breast** | 482 | | 0.386 (0.353-0.422) | | 0.99 (0.91-1.08) | 5 | 57 | 2.15 (0.84-5.46) | 0.1 |

^1^ Rate ratios calculated by Poisson regression adjusted for age group and period

**Supplementary Table 3: Cumulative risk of invasive cervical cancer by HPV type and age at baseline to the Manchester Cohort (n=6,215) and the ARTISTIC Cohort (n=24,496). Women are censored at the first occurrence of ICC, but not by age^1^**

| **Status at baseline** |  |  | **5 year risk from baseline** | | | **15 year risk from baseline** | | | **30 year risk from baseline** | | |
| --- | --- | --- | --- | --- | --- | --- | --- | --- | --- | --- | --- |
|  | **n (%) at baseline** | **n ICC^2^** | **n ICC** | **%** | **95% CI** | **n ICC** | **%** | **95% CI** | **n ICC** | **%** | **95% CI** |
| **Manchester Cohort** |  |  |  |  |  |  |  |  |  |  |  |
| HR HPV + at baseline |  |  |  |  |  |  |  |  |  |  |  |
| HPV 16/HPV 18 | 270 (4.3%) | 8 | 2 | 0.74% | (0.19 – 2.93) | 5 | 1.87% | (0.78 – 4.43) | 8 | 3.10% | (1.56 – 6.11) |
| Other HR HPV | 155 (2.5%) | 2 | 1 | 0.65% | (0.09 – 4.49) | 2 | 1.31% | (0.33 – 5.14) | 2 | 1.31% | (0.33 – 5.14) |
| Age at baseline: <20 | 65 (15.3%) | 2 | 0 |  |  | 2 | 3.08% | (0.78-11.75) | 2 | 3.08% | (0.78-11.75) |
| 20-39 | 291 (68.5%) | 5 | 1 | 0.34% | (0.05 - 2.41) | 3 | 1.04% | (0.34 - 3.20) | 5 | 1.79% | (0.75 - 4.24) |
| 40-69 | 69 (16.2%) | 3 | 2 | 2.92% | (0.74 – 11.18) | 2 | 2.92% | (0.74 – 11.18) | 3 | 4.86% | (1.57 – 14.49) |
| HR HPV - at baseline |  |  |  |  |  |  |  |  |  |  |  |
| Age at baseline: <20 | 262 (4.5%) | 0 |  |  |  |  |  |  |  |  |  |
| 20-39 | 2942 (50.8%) | 5 | 1 | 0.03% | (0.00 – 0.24) | 3 | 0.10% | (0.03 – 0.32) | 5 | 0.18% | (0.07 – 0.42) |
| 40-69 | 2586 (44.7%) | 3 | 0 |  |  | 1 | 0.04% | (0.01 – 0.29) | 3 | 0.17% | (0.05 – 0.58) |
| **ARTISTIC Cohort** |  |  |  | | |  | | |  |  |  |
| HR HPV + at baseline |  |  |  |  |  |  |  |  |  |  |  |
| HPV 16^6^ | 778 (3.2%) | 9 | 4 | 0.51% | (0.19-1.36) | 8 | 1.03% | (0.52-2.05) |  |  |  |
| HPV18^6^ | 326 (1.4%) | 2 | 1 | 0.31% | (0.04-2.16) | 2 | 0.61% | (0.15-2.43) |  |  |  |
| Other HR HPV | 1,676 (6.8%) | 8 | 4 | 0.24% | (0.09-0.63) | 8 | 0.48% | (0.24-0.95) |  |  |  |
| Age at baseline: 20-39 | 2,341 (83.5%) | 11 | 4 | 0.17% | (0.06-0.45) | 10 | 0.43% | (0.23-0.79) |  |  |  |
| 40-64 | 463 (16.5%) | 9 | 6 | 1.30% | (0.58-2.86) | 9 | 1.95% | (1.02-3.71) |  |  |  |
| HR HPV - at baseline |  |  |  |  |  |  |  |  |  |  |  |
| Age at baseline: 20-39 | 10,486 (48.3%) | 7 | 2 | 0.02% | (0.00-0.08) | 4 | 0.04% | (0.01-0.10) |  |  |  |
| 40-64 | 11,224 (51.7%) | 5 | 0 |  |  | 5 | 0.04% | (0.02-0.11) |  |  |  |

^1^ One cervical cancer diagnosed age 88 in the MC appears in this table but is censored in table 1

^2^ to the end of follow-up to March 2019 (max follow-up 30 years for MC and 17 years for AC)

^3^ tested HPV positive with general primer but negative for HR type-specific primers.

^4^ includes 24 women who were HC2+ and insufficient for typing (includes 1 diagnosed with ICC at baseline following high grade cytology)

^5^ HC2 negative and HC2 positive but with no HR-HPV DNA detected on genotyping.

^6^ HPV16/HPV18 combined 15 year cumulative risk = 0.91% (0.49-1.68)

**Supplementary Table 4a: Cumulative risk of CIN3 by HPV type and age at baseline of the Manchester Cohort. Women are censored at the first occurrence of CIN3.**

| **Status at baseline** |  |  | **5 year risk from baseline** | | | **15 year risk from baseline** | | | **30 year risk from baseline** | | |
| --- | --- | --- | --- | --- | --- | --- | --- | --- | --- | --- | --- |
|  | **n (%) at baseline** | **n CIN3^1^** | **n CIN3** | **%** | **95% CI** | **n CIN3** | **%** | **95% CI** | **n CIN3** | **%** | **95% CI** |
| **HR HPV + at baseline:** |  |  |  |  |  |  |  |  |  |  |  |
| **HPV 16** | 206 (3.3%) | 37 | 25 | 12.19% | (8.41 – 17.51) | 37 | 18.18% | (13.52 – 24.20) | 37 | 18.18% | (13.52 – 24.20) |
| **HPV 18** | 64 (1.0%) | 5 | 1 | 1.56% | (0.22 – 10.58) | 5 | 8.02% | (3.42 – 18.21) | 5 | 8.02% | (3.42 – 18.21) |
| **Other HR HPV** | 155 (2.5%) | 7 | 6 | 3.89% | (1.77 – 8.45) | 7 | 4.56% | (2.20 – 9.32) | 7 | 4.56% | (2.20 – 9.32) |
|  |  |  |  |  |  |  |  |  |  |  |  |
| **Age at baseline: <20** | 65 (15.3%) | 6 | 3 | 4.62% | (1.51 – 13.63) | 6 | 9.28% | (4.28 – 19.50) | 6 | 9.28% | (4.28 – 19.50) |
| **20-24** | 87 (20.5%) | 5 | 2 | 2.30% | (0.58 – 8.88) | 5 | 5.75% | (2.43 – 13.26) | 5 | 5.75% | (2.43 – 13.26) |
| **25-29** | 86 (20.2%) | 15 | 10 | 11.67% | (6.46 – 20.61) | 15 | 17.58% | (10.99 – 27.46) | 15 | 17.58% | (10.99 – 27.46) |
| **30-39** | 118 (27.8%) | 14 | 10 | 8.51% | (4.67 – 15.24) | 14 | 11.98% | (7.28 – 19.40) | 14 | 11.98% | (7.28 – 19.40) |
| **40-69** | 69 (16.2%) | 9 | 7 | 10.24% | (5.02 – 20.28) | 9 | 13.50% | (7.25 – 24.38) | 9 | 13.50% | (7.25 – 24.38) |
|  |  |  |  |  |  |  |  |  |  |  |  |
| **HR HPV - at baseline** |  |  |  |  |  |  |  |  |  |  |  |
| **Age at baseline: <20** | 262 (4.5%) | 13 | 1 | 0.38% | (0.05 – 2.68) | 10 | 3.91% | (2.12 – 7.14) | 13 | 5.17% | (3.03 – 8.75) |
| **20-24** | 395 (6.8%) | 18 | 3 | 0.76% | (0.25 – 2.34) | 11 | 2.80% | (1.56 – 5.01) | 18 | 4.64% | (2.95 – 7.26) |
| **25-29** | 536 (9.3%) | 10 | 3 | 0.56% | (0.18 – 1.73) | 9 | 1.69% | (0.88 – 3.23) | 10 | 1.89% | (1.02 – 3.48) |
| **30-39** | 2011 (34.7%) | 29 | 10 | 0.50% | (0.27 – 0.93) | 26 | 1.31% | (0.90 – 1.92) | 29 | 1.51% | (1.05 – 2.17) |
| **40-49** | 1403 (24.2%) | 7 | 2 | 0.14% | (0.04 – 0.57) | 7 | 0.51% | (0.24 – 1.06) | 7 | 0.51% | (0.24 – 1.06) |
| **50-69** | 1183 (20.4%) | 3 | 2 | 0.17% | (0.04 – 0.68) | 3 | 0.26% | (0.08 – 0.81) | 3 | 0.26% | (0.08 – 0.81) |
|  |  |  |  |  |  |  |  |  |  |  |  |

^1^ to the end of follow-up to March 2019 (max follow-up 30 years)

**Supplementary Table 4b – supplementary material: Cumulative risk of CIN3 by HPV type and age at baseline of ARTISTIC trial**

| **Status at baseline** |  |  | **5 year risk from baseline** | | | **15 year risk from baseline** | | |
| --- | --- | --- | --- | --- | --- | --- | --- | --- |
|  | **n (%) at baseline** | **n CIN3^1^** | **n CIN3** | **%** | **95% CI** | **n CIN3** | **%** | **95% CI** |
| **HR HPV + at baseline:** |  |  |  |  |  |  |  |  |
| **HPV 16** | 778 (3.2%) | 180 | 159 | 21.04% | (18.30-24.13) | 179 | 23.86% | (20.96-27.09) |
| **HPV 18** | 326 (1.3%) | 29 | 29 | 9.13% | (6.43-12.87) | 39 | 12.35% | (9.18-16.52) |
| **Other HR HPV** | 1676 (6.8%) | 115 | 83 | 4.98% | (4.04-6.14) | 111 | 6.70% | (5.60-8.02) |
|  |  |  |  |  |  |  |  |  |
| **Age at baseline: 20-24** | 875 (31.2%) | 104 | 74 | 8.56% | (6.88-10.63) | 101 | 11.75% | (9.77-14.10) |
| **25-29** | 611 (21.8%) | 83 | 67 | 11.22% | (8.94-14.04) | 81 | 13.62% | (11.10-16.64) |
| **30-39** | 855 (30.5%) | 114 | 98 | 11.59% | (9.61-13.94) | 113 | 13.41% | (11.28-15.90) |
| **40-64** | 463 (16.5%) | 35 | 33 | 7.23% | (5.20-10.02) | 35 | 7.71% | (5.60-10.58) |
|  |  |  |  |  |  |  |  |  |
| **HR HPV - at baseline** |  |  |  |  |  |  |  |  |
| **Age at baseline: 20-24** | 1721 (7.9%) | 35 | 4 | 0.23% | (0.09-0.62) | 30 | 1.75% | (1.23-2.49) |
| **25-29** | 1975 (9.1%) | 17 | 5 | 0.25% | (0.11-0.61) | 15 | 0.76% | (0.46-1.26) |
| **30-39** | 6772 (31.2%) | 25 | 7 | 0.10% | (0.05-0.22) | 23 | 0.34% | (0.23-0.51) |
| **40-49** | 5795 (26.7%) | 8 | 3 | 0.05% | (0.02-0.16) | 6 | 0.10% | (0.05-0.23) |
| **50-64** | 5429 (25.0%) | 3 | 2 | 0.04% | (0.01-0.15) | 1 | 0.07% | (0.02-0.23) |
|  |  |  |  |  |  |  |  |  |

^1^ to the end of follow-up to end March 2019 (max follow-up 17.8 years). 93 CIN3s with laboratory registration to end 2009 without national registration were included

^2^ includes 24 women who were HC2+ and insufficient for typing (includes 1 diagnosed with CIN3 following high grade cytology)

^3^ HC2 negative and HC2 positive but with no HR-HPV DNA detected on genotyping.
